# Altered chromatin accessibility in light and hormone responses of rice seedlings

**DOI:** 10.1093/plphys/kiaf438

**Published:** 2025-09-26

**Authors:** Haoxuan Li, Xiaozheng Li, Jianhua Zhang, Guanqun Wang

**Affiliations:** School of Life Sciences, AoE Centre for Plant Vacuole Biology and Biotechnology, and State Key Laboratory of Agrobiotechnology, The Chinese University of Hong Kong, Shatin, Hong Kong; College of Life Sciences and Oceanography, Shenzhen University, Shenzhen 518054,China; Department of Biology, Hong Kong Baptist University, Hong Kong; College of Life Sciences and Oceanography, Shenzhen University, Shenzhen 518054,China; School of Life Sciences, AoE Centre for Plant Vacuole Biology and Biotechnology, and State Key Laboratory of Agrobiotechnology, The Chinese University of Hong Kong, Shatin, Hong Kong; Department of Biology, Hong Kong Baptist University, Hong Kong; School of Life Sciences, AoE Centre for Plant Vacuole Biology and Biotechnology, and State Key Laboratory of Agrobiotechnology, The Chinese University of Hong Kong, Shatin, Hong Kong

## Abstract

Light is essential for plant adaptation and the survival of photoautotrophs. However, our understanding of how rice (*Oryza sativa*) seedlings transition from skotomorphogenesis to photomorphogenesis remains limited, especially at the epigenetic level. Here, we show that light greatly alters chromatin accessibility during the switch from skotomorphogenesis to photomorphogenesis. Photosynthesis-related genes exhibited more open chromatin, although more closed chromatin was identified after light exposure at the genome-wide scale. These largely closed chromatin structures parallel the reduced transcriptional activity revealed by RNA polymerase II (Pol II) occupancy. Moreover, transcription activators of GOLDEN2-LIKE (GLKs), which control chloroplast biogenesis and development, primarily bind to light-induced open chromatin regions, thereby functionally establishing rice as a photoautotroph. Additionally, the integrated analysis of chromatin accessibility and Pol II occupancy in response to exogenous indole-3-acetic acid (IAA) and abscisic acid (ABA) application revealed that IAA and ABA have active and repressive roles in inducing chromatin openness and transcriptional activity in rice seedlings, respectively. Collectively, our results provide insight into the epigenomic regulation of rice seedling photomorphogenesis and valuable resources for studying the roles of cis-regulatory elements in the regulation of hormone responses, specifically IAA and ABA, in rice seedlings.

## Teaser

Light greatly alters chromatin accessibility during the switch from skotomorphogenesis to photomorphogenesis in rice.

## Introduction

Light transmits key information about the environment, which drives seedlings from skotomorphogenesis to photomorphogenesis and subsequently causes the inhibition of hypocotyl elongation, cotyledon opening and expansion, and maturation of green chloroplasts for photosynthesis. Therefore, light is crucial for plant adaptation and photoautotroph. *Arabidopsis* seedling photomorphogenesis has been well established. Multiple photoreceptors have been identified to be involved in the response of wavelengths of light, light direction, and light duration. These include blue/ultraviolet (UV)-A light photoreceptors cryptochromes [CRYs (Cashmore et al. 1999)] and phototropins (Briggs and Christie 2002) and ZTL/FKF1/LKP2 (Ito et al. 2012), red/far-red light (RL/FRL) photoreceptors phytochromes (Quail 2002), and UV-B light photoreceptor UVB-RESISTANCE 8 (Rizzini et al. 2011). Rice seedlings grown in the dark develop long coleoptiles while undergoing regular circumnutation, showing the characteristic skotomorphogenesis. However, the understanding on how monocotyledon plants, such as rice, go through the switches from skotomorphogenesis to photomorphogenesis remains largely unknown.

Plant development is driven by precise gene expression patterns that largely accomplished through coordinated binding of regulatory proteins to cis-regulatory elements within promoters, enhancers, and other cis-regulatory elements. Active cis-regulatory elements located in the genomic regions are open to regulatory proteins, resulting in gene transcription (Yan et al. 2019; Han et al. 2020). Genome-wide open chromatin mapping enables the identification of the cis-regulatory elements both in genic and intergenic regions (Rodgers-Melnick et al. 2016; Yan et al. 2019; Han et al. 2020). For example, assays of transposase accessible chromatin sequencing (ATAC-seq), have been successfully deployed to generate large-scale open chromatin maps in plants (Wang et al. 2020a; Wang et al. 2022), which contribute to insightful information of the regulation of gene expression in the biological basis at the epigenetic level (Klemm et al. 2019). Plant nuclear hormone signaling elicits diverse developmental responses in different cell types. Auxin, one of the most prominent hormones, functions throughout plant development, acting embryonically, postembryonically, and above and below ground (Moller et al. 2017). Abscisic acid (ABA) acts as a sensitive signal during seed germination, drought stress, grain filling, photosynthesis, and other developmental stages (Wang et al. 2019). However, how the chromatin accessibility correlated with treatments of exogenous indole-3-acetic acid (IAA) and ABA in rice seedlings is still obscure in rice.

To provide better understanding of rice seedling transition from skotomorphogenesis to photomorphogenesis at both epigenetic and transcriptional levels, we applied ATAC-seq, RNA-seq, and ChIP-seq to elucidate chromatin accessibility, transcriptomes, and transcription activity. We find that light rapidly established seedling photomorphogenesis, including switched chloroplast development, by altering chromatin accessibility and transcriptional activity. The analysis of chromatin accessibility and GOLDEN2-LIKE (GLK) transcription factor (TF) ChIP-seq further reveals the roles of rice GLKs in controlling chloroplast biogenesis and development, subsequently underlying photomorphogenesis establishment in the epigenetic level. Moreover, the chromatin accessibility and transcriptional activity greatly activated in response to exogenous auxin, while its response to ABA was not that pervasive. Overall, our results revealed a comprehensive understanding of rice seedling photomorphogenesis in the epigenetic level and provided valuable resources for studying the roles of cis-regulatory elements in hormone responses in rice seedling.

## Results

### Overview of chromatin accessibility during rice seedling photomorphogenesis

**Figure 1.**
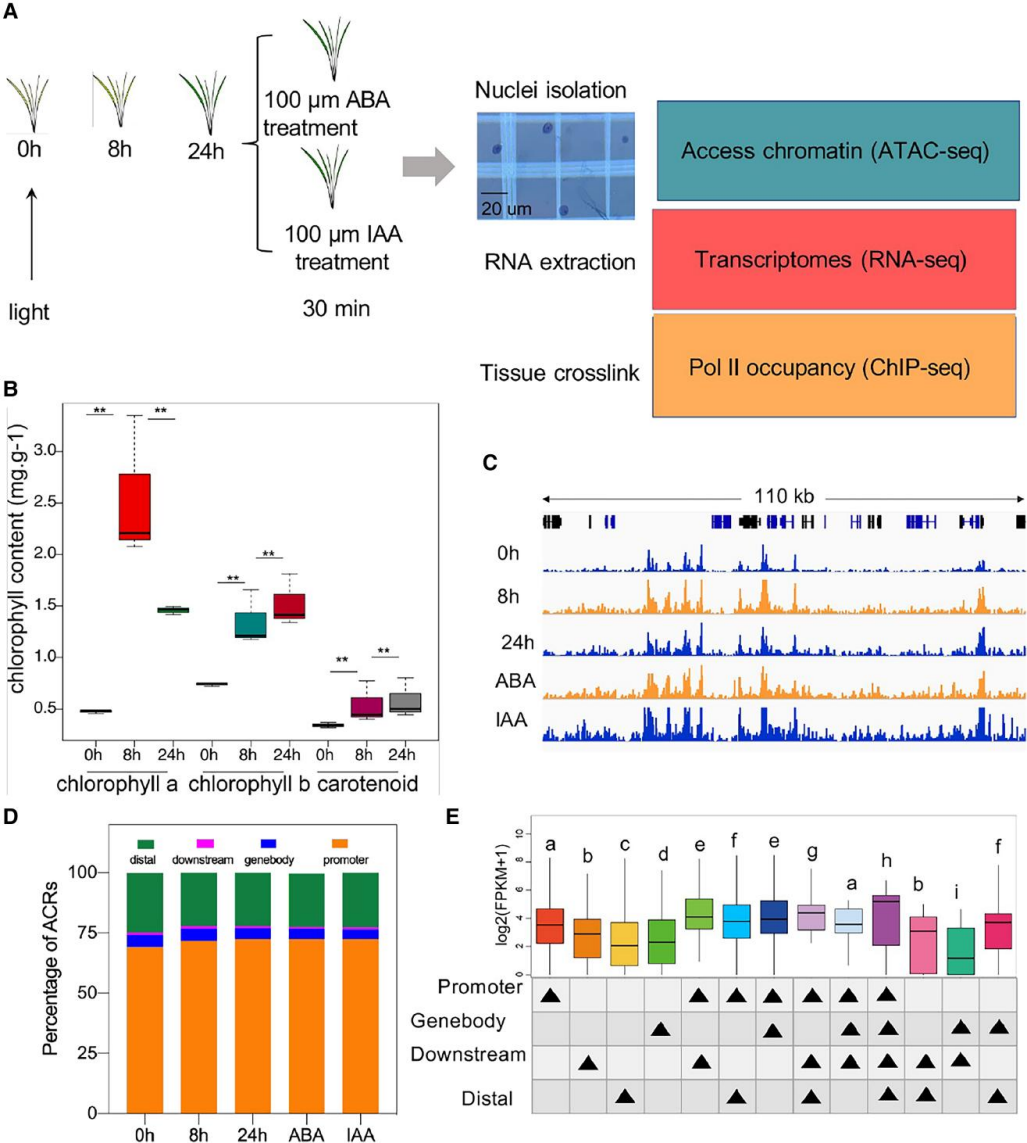
Combination of ATAC-, RNA-, and ChIP-seq to study chromatin accessibilities, gene expressions, and Pol II occupancy during rice seedling photomorphogenesis. **A)** Schematic outline of genome-wide ATAC-seq, RNA-seq, and ChIP-seq assays and time points of sample collections. ABA and IAA were sprayed to the seedlings at 24 h to investigate the chromatin accessibility in response to exogenous hormone. The quality of nuclei was validated with trypan blue staining; the scale bar is shown. **B)** Chlorophyll content of Chlorophyll a, Chlorophyll b, and carotenoid measured at 0, 8 and 24 h. **C)** Integrative Genomics Viewer showing ATAC-seq signals in a 110 kb region (Chr3:26,020,000–26,130,000) in 5 samples as indicated. Tracks showed normalized values of 1 representative biological replicate for each sample. **D)** The percentage of ACRs corresponding to categories of promoter (TSS upstream 2 kb), gene body, downstream (TES downstream 300 bp), and distal intergenic for 0, 8, and 24 h, ABA and IAA. **E)** Boxplots showing expression levels of genes associated with promoter, gene body, downstream, and distal intergenic ACRs. Boxplots show the median (horizontal line), second to third quartiles (box), and Tukey style whiskers (beyond the box). Statistical significance was calculated based on pairwise Wilcoxon signed-rank tests. *P < 0.05; **P < 0.01.

After seed germination, seedling photomorphogenesis is one of the earliest responses to light in higher plants. To provide a comprehensive understanding of rice seedling photomorphogenesis, the rice seedling germinated under dark for 3 d were then exposed to light. Next, we sampled the seedlings under dark as the first time point (0 h) and seedlings exposed to light for 8 h (8 h) and 24 h (24 h) for the second and third time points, respectively. In addition, to understand how chromatin accessibility correlated with exogenous auxin and ABA, we performed short-term auxin (IAA) and ABA treatment on rice seedlings (100 *μ*ᴍ IAA and 100 *μ*ᴍ ABA for 30 min) (Fig. 1A). To profile the light responses of rice seedling in the physiological level, we measured the Chlorophyll a and b and carotenoid content across the 3 time points (Fig. 1B). Results showed that Chlorophyll a displayed dramatic increase from 0 to 8 h, while a sharp reduction was found at 24 h. In contrast, Chlorophyll b and carotenoid gradually increased after light exposure. These results suggested dramatic chloroplast development and photosynthesis during photomorphogenesis.

Chromatin accessibility determines the functional state of a cell. To provide a comprehensive understanding of chromatin accessibility during rice seedling photomorphogenesis, we constructed genome-wide maps of ATAC-seq on rice seedlings at 0, 8 and 24 h after light treatment. To identify accessible chromatin regions (ACRs), ATAC-seq reads were analyzed by using MACS2 peak calling software. Overlapped peaks called in the 2 biological replicates of each time point were considered as ACRs (Supplementary Table S1). A genome browser overview of an exemplary 110 kb genomic region illustrates the occurrence of enriched signals as sharp peaks in all samples (Fig. 1C). The principal component analysis (PCA) comparing the enrichment values at all ACRs across all samples showed that biological replicates clustered closely together (Supplementary Fig. S1A). ACRs were widely distributed throughout the genome, with highest enrichment at transcription start sites (TSSs) and mild enrichment at transcription termination sites (TTSs) (Supplementary Fig. S1B). We further explored the ACR distribution across the whole genome by assigning each ATAC-seq peak to the nearest gene based on its annotated TSS. Most of the ACRs (∼70%) were localized at <2 kb upstream of the TSS, hereafter referred to as promoter region, which followed by ACRs at gene distal or intergenic regions (∼22%), gene body regions (∼5%), and downstream regions (<300 bp downstream of the TTS, ∼1%). We found that light increased the proportion of ACRs distributed in the promoter regions accompanied with reduced ACR percentage in distal regions (Fig. 1D). To explore the correlation between ACRs and transcript, we also performed RNA-seq of each sample. By integrating the chromatin accessibility data with the RNA-seq data, we found that the expression levels of genes associated with only the promoter region was significantly higher than those only associated with the gene body, downstream or distal regions, while genes associated with more than 1 ACR category expressed overall at higher levels than those associated with fewer (Fig. 1E).

### Diverse light-responsive chromatin accessibility patterns during rice seedling photomorphogenesis

**Figure 2.**
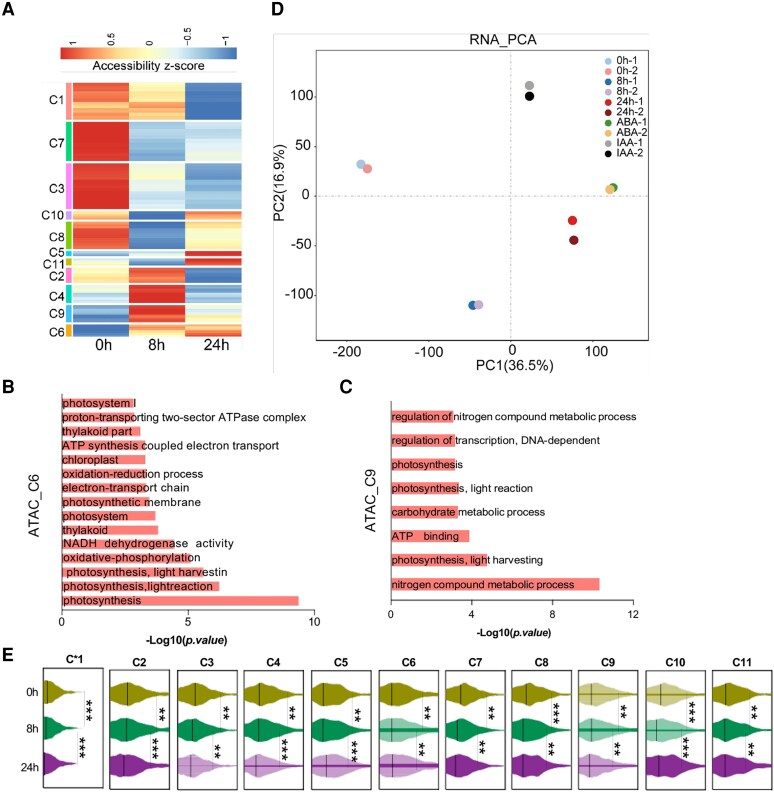
Overview of chromatin accessibility and transcriptome dynamics during photomorphogenesis. **A)** Heatmap of differentially accessible peaks (fold change > 1.5) sorted by K-means clustering across the samples collected at different time points (0, 8, and 24 h). Color bar, accessibility Z score of differentially accessible peaks identified by ATAC-seq. **B** and **C)** GO analysis of Clusters C6 and C9. **D)** PCA plots of RNA-seq data. Color code is shown. Each dot represents 1 sample. **E)** Transcription levels associated with each ATAC cluster were shown. The median value (horizontal line) was shown in each violin plot. Statistical significance was calculated based on pairwise Wilcoxon signed-rank tests. *P < 0.05; **P < 0.01; ***P < 0.001.

To achieve the light-responsive chromatin accessibility pattern, we explored K-means clustering approach based on the level of chromatin accessibility to sort all differential peaks resulting in 11 clusters, named C1 to C11, respectively (Fig. 2A and Supplementary Table S2). The sites in Clusters C1, C3, and C7 displayed opened chromatin at 0 h and lost chromatin accessibility after light exposure. Clusters C8, C10, and C11 first lost accessibility at 8 h, and then the chromatin accessibility increased at 24 h. The sites in Clusters C5 and C6 were closed under dark at 0 h and gradually open its chromatin under light at 8 and 24 h. The genes responsible for the photosynthesis process was identified in C6 cluster by gene ontology (GO) enrichment analysis (Fig. 2B). In contrast, the genes belonging to Clusters C2, C4, and C9 showed opened chromatin at 8 h and closed chromatin at 24 h. GO analysis suggested that photosynthesis related genes were also enriched in cluster C9 (Fig. 2C). Interestingly, we observed a significantly higher number of closed chromatin regions compared to those opened chromatin regions, indicating the overall inactive transcription activity during photomorphogenesis. Collectively, the above results reveal that the chromatin accessibility landscape of seedling undergoes rapid and massive transitions during photomorphogenesis.

Next, we analyzed RNA-seq data of samples collected at the same time points. PCA showed high concordance between replicates (Fig. 2D and Supplementary Table S3). The expression levels of genes associated with ACRs belonging to the above 11 ACR clusters were analyzed. The results showed that the transcript levels of genes associated with C1, C3, and C7 clusters changed similar to that of chromatin accessibility, which gradually decreased from 0 to 24 h (Fig. 2E). In addition, Clusters C2, C5, C8, and C10 also showed similar trend to that of chromatin accessibility (Fig. 2E); for instance, the gene of LOC_Os02g03670 belonged to Cluster C2, and the gene of LOC_Os02g30110 to Cluster C5 (Supplementary Fig. S2, A and B). To further explore the correlation between transcript levels and chromatin accessibility, we compared the differential ACRs (DACRs) with differentially expressed genes (DEGs). However, we found that only 37% DEGs were correlated with DACRs in 8 h versus 0 h, while 26% DEGs identified in 24 versus 8 h were correlated with DACRs (Supplementary Fig. S2, C to F). This probably revealed that chromatin accessibility is not always correlated with its expression level, which was also being determined by DNA methylation levels and the type of histone modifications in the promoter (Klemm et al. 2019). Since TFs probably display binding preferences at DACRs, we performed ATAC-seq footprint analysis (Bentsen et al. 2020) for genome-wide TF motif scanning (Supplementary Table S4). As observed, some TF binding motifs showed significantly different ATAC-seq footprint scores after exposure to light conditions, indicating that these TFs might play important roles in forming the DACRs (Supplementary Table S5).

### GLKs contribute to rice photomorphogenesis through binding on light-responsive opened chromatin regions

**Figure 3.**
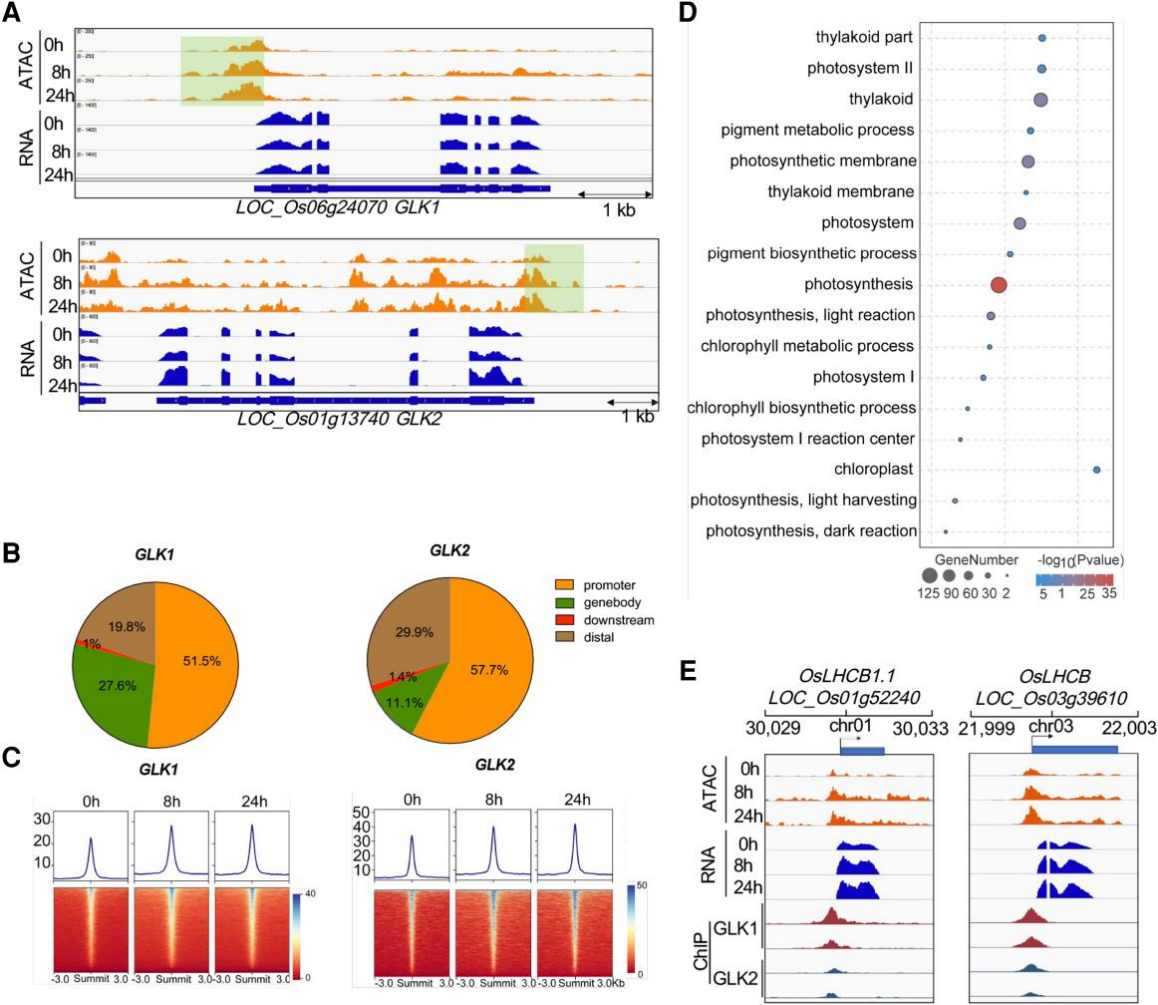
GLKs bind to opened ACRs to control chloroplast biogenesis and development. **A)** The ATAC-seq tracks (upper panel) and RNA-seq data (lower panel) for 2 GLKs. The genomic regions are shown, and the location of differential peaks is shadowed. **B)** Distributions of GLK ChIP-seq peaks in the promoter, gene body, downstream, and distal regions. **C)** ChIP-seq signal of 2 GLK genes enriched at the ACR peak summit. Regions of ±3 kb from peak summits were shown. **D)** GO enrichment analysis of GLK2 peak-associated genes. **E)** Representative targets of GLKs that are involved in the photosynthesis process. Light harvest genes, for instance, *OsLHCB1.1* (*LOC_Os01g52240*) and *OsLHCB* (*LOC_Os03g39610*).

Photosynthesis is arguably one of the most important and conserved biological processes in plants, and GOLDEN2-LIKE (GLK) are well-known transcription activators controlling chloroplast biogenesis and development (Tu et al. 2022). Two redundant copies of GLK exist in rice genome. We would like to know how chromatin accessibility coordinates with GLK binding to control gene expression during chloroplast biogenesis in response to light. To this end, we first explored the chromatin accessibility and gene expression levels of 2 GLK genes. Gradually opened chromatin accessibility was identified in both GLK1 and GLK2 during the switches from dark to light. Both GLK1 and GLK2 were highly expressed across the 3 stages even under the dark conditions (Fig. 3A). The expression levels of GLK1 maintain a similar level across the 3 stages. In contrast, GLK2 was significantly induced by light under 24 h (Fig. 3A). Next, we asked about the correlation between ACRs and GLK binding sites in rice. To answer this, we thereby retrieved 2 GLKs' ChIP-seq datasets of rice (Tu et al. 2022) and analyzed its binding sites (Fig. 3B). We found that more than 50% of the 2 GLK binding sites were distributed in the promoter region, followed by distal and gene body regions (Fig. 3B). By comparing the GLK peaks to ACRs, we found that the signals of the 2 GLKs ChIP-seq datasets were enriched at the ACR peak centers (Fig. 3C). Moreover, the intensity of those binding sites increased under light, suggesting that light increased chromatin accessibility of GLK targeted genes, thus allowing GLK binding on these ACRs. We also annotated the binding sites of 2 GLKs to its nearby genes and performed the GO enrichment analysis. As expected, the GLK-targeted genes were significantly enriched in the photosynthesis-related genes, for example, the chlorophyll biosynthetic process, pigment biosynthetic process, thylakoid, and photosystem (Fig. 3D). Then individual gens, such as the chlorophyll-binding proteins LIGHT HARVESTING COMPLEX A/B (LHCA/B) and those encoding subunits of Photosystem I/II, showed 5′ end proximal GLK1 and GLK2 ChIP-seq peaks (Fig. 3E and Supplementary Fig. S3). These peaks also overlapped with the increased ATAC-seq peaks under light (Fig. 3E and Supplementary Fig. S3), which were accompanied with increased transcript levels. These results suggested that GLKs bind to the opened chromatin regions on the promoter of photosynthetic-related genes, promoting to build photosynthesis under light.

### The roles of auxin and ABA in regulation of gene expression through the epigenetic levels in rice seedling

**Figure 4.**
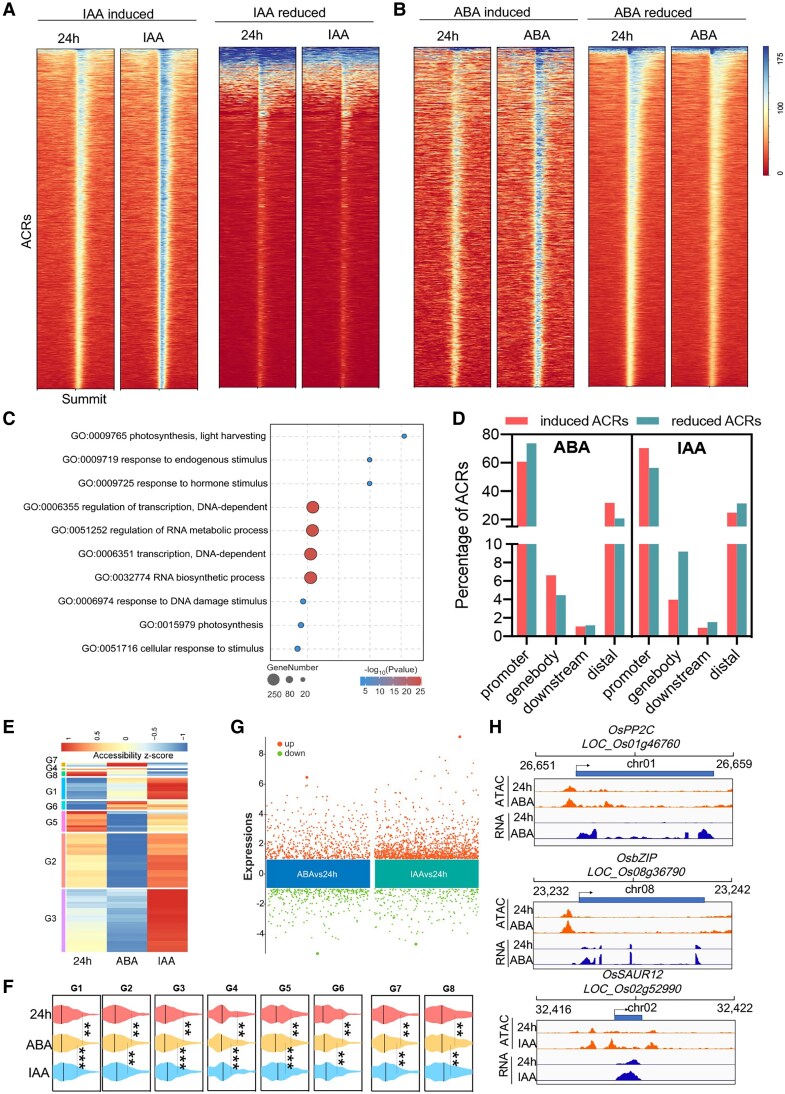
Characterization of ABA-/IAA-induced or IAA-repressed ACRs. **A** and **B)** Heatmaps showing ATAC-seq coverage from −2 to +2 kb around the center of IAA-altered (including induced and repressed) **(A)** and ABA-altered (including induced and repressed) ACRs **(B)**. **C)** GO analysis showing enriched pathways of genes associated with ABA-repressed ACRs. **D)** Bar plots showing percentage of responsive ACRs per ACR category. **E)** Heatmap of differentially accessible peaks (fold change > 1.5) sorted by K-means clustering across the samples after treatment with ABA or IAA. Color bar, accessibility *Z* score of differentially accessible peaks identified by ATAC-seq. **F)** Transcript levels of those genes associated with differentially accessible peaks within different clusters. **G)** Scatter plot showing up- or downregulated genes regulated by ABA and IAA. Upper dots showing the downregulated genes, while lower dots showing the upregulated genes. **H)** Examples of the positive correlation between chromatin accessibility and transcript levels regulated by ABA or IAA. OsPP2C and OsbZIP altered by ABA were responsible for ABA signal transduction, while IAA changed the chromatin state and transcript levels of OsSAUR12. Statistical significance was calculated based on pairwise Wilcoxon signed-rank tests. *P < 0.05; **P < 0.01; ***P < 0.001.

To understand the dynamic chromatin accessibility of rice seedling responding to hormones of IAA and ABA, we performed short-term auxin or ABA treatment on rice seedlings followed by ATAC-seq and RNA-seq, respectively. If an ACR showed increased or decreased tagment signals >1.5 fold after the treatment of IAA or ABA, it was considered as induced and repressed ACR. Overall, we identified 8,331 IAA-induced ACRs and 1,820 IAA-reduced ACRs, suggesting the predominant role of auxin in activating the chromatin to open (Fig. 4A). Regarding ABA responses, we identified 1,318 ABA-induced ACRs and 9,827 ABA-repressed ACRs. This might suggest the role of ABA in repressing chromatin accessibility (Fig. 4B). Thereby, auxin-responsive chromatin accessibility was opposite to that of ABA. Genes associated with ABA and IAA altered ACRs enriched in several GO categories. Interestingly, genes associated with ABA-repressed ACRs showed significantly enriched biological pathways of response to hormone stimulus and response to endogenous stimulus and photosynthesis, indicating the inhibition of photosynthesis by ABA (Fig. 4C). Genes associated with ABA-induced ACRs are enriched in the nitrogen compound metabolic process and gene expression pathways (Supplementary Fig. S4A). Genes associated with IAA-induced ACRs were significantly enriched in the pathways of nucleic acid binding and transcription regulator activity (Supplementary Fig. S4B). In contrast, genes associated with IAA-repressed ACRs were greatly enriched in photosynthetic-related pathways (Supplementary Fig. S4C). In addition, we evaluated whether ACRs on different genomic categories showed a different response to ABA/auxin. Through the comparisons, we observed that the fractions of ABA-induced ACRs in the promoter and downstream regions were lower than those of ABA-reduced ACRs, while fractions of ABA-induced ACRs in the gene body and distal regions were much higher than those of ABA-reduced ACRs (Fig. 4D). In contrast, the fractions of IAA-induced promoter ACRs were higher than IAA-reduced ACRs, whereas IAA-reduced ACRs in the gene body, downstream, and distal regions were much less than those of IAA-reduced ACRs (Fig. 4D).

Next, we explored the K-means clustering approach based on the level of chromatin accessibility to sort all ABA- and IAA-altered peaks resulting in 8 clusters, named G1 to G8, respectively (Supplementary Table S6). The results also showed that most of the ABA-responsive ACRs were closed, while auxin-responsive ACRs were more opened (Fig. 4E). Moreover, gene expression pattern further demonstrated the reduced transcript levels in ABA-responsive ACR-associated genes (Fig. 4F). We also compared the DEGs caused by ABA and auxin, respectively (Fig. 4G). The number of the ABA-upregulated genes (1,676) and ABA-downregulated genes (1,654) were similar, while the number of the total DEGs was much less than these DACRs (Fig. 4G). In terms of auxin-altered DEGs, a total of 3,204 genes were upregulated, while 1,218 genes were downregulated (Fig. 4G). For example, ABA-responsive DEGs involved in the ABA signaling pathway encoding PP2C and bZIP were induced by ABA, which was accompanied with increased chromatin accessibility (Fig. 4H). OsSAUR12 gene involved in auxin signaling was upregulated, consisting with elevated chromatin accessibility under auxin treatment (Fig. 4H). Additionally, our ATAC-seq footprint analysis (Bentsen et al. 2020) for TF motif scanning (Supplementary Table S4) revealed a large number of TF binding motifs that showed significantly different ATAC-seq footprint scores after the ABA treatment and the IAA treatment (Supplementary Table S5), respectively, suggesting that hormone-responsive TFs are critical for the DACR formation.

### Light reduces transcription activity and IAA activates gene transcription in rice

**Figure 5.**
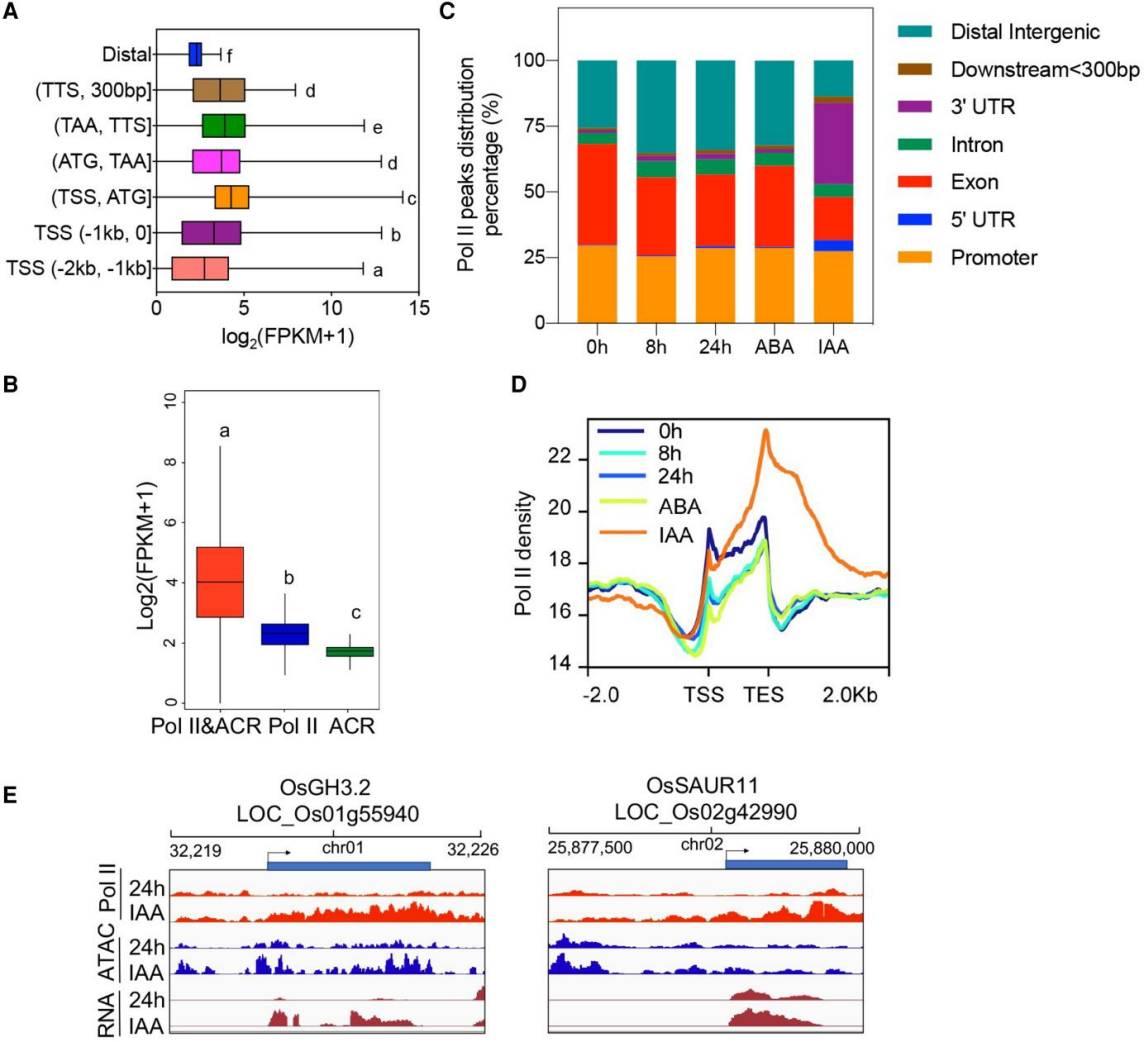
RNA Pol II occupancy during rice photomorphogenesis and its response to auxin and ABA. **A)** Boxplots showing expression levels of genes associated with RNA Pol II occupancy in different regions of the genome structure. TSS, transcription start sites; TTS, transcription termination site. ATG is the start codon, and TAA is the stop codon. Boxplots show the median (horizontal line). Upper and lower quartiles were the box limits. The outlier is the data point that is located outside the whiskers of the boxplot. Different letters represent significant difference. **B)** The transcription activity of genes associated with both Pol II and ACR, genes associated with non-Pol II-modified ACR, and genes associated with only Pol II modification. Boxplots show the median (horizontal line). Upper and lower quartiles were the box limits. The outlier is the data point that is located outside the whiskers of the boxplot. Different letters represent significant difference. FPKM, fragments per kilobase per million. **C)** Pol II peak distribution on different gene structures. UTR, untranslated region. **D)** Pol II density among all the samples and IAA treatment dramatically altered Pol II profile in the gene coding region. TSS, transcription start sites; TES, transcription end sites. **E)** Exogenous IAA-induced ACRs associated with auxin signal transduction, allowed the Pol II binding, and subsequently promoted its transcript levels. Pol II, RNA polymerase II.

In eukaryotic cells, protein-coding genes, as well as some noncoding genes, are transcribed by DNA-dependent RNA polymerase II (Pol II), which executes a series of distinct steps: including binding to promoters, initiating RNA synthesis, and then pausing in early transcriptional elongation. To profile genome-wide transcriptional dynamics and transcribing genes during rice photomorphogenesis, RNA Pol II ChIP-seq was conducted. Then, we plotted the correlation between Pol II occupancy and pattern of gene expressions using samples of 0 h as an example. As observed, genes with Pol II binding from regions of TSS to ATG displayed the highest expression levels among all the other Pol II occupancy, which is followed by Pol II occupancy from TAA to TTS, and gene body regions (Fig. 5A). Next, we explored the roles of Pol II binding in transcription by classifying these genes occupied with dual modifications, including both Pol II and ACR, occupied only with Pol II and only featured with ACR, respectively (Fig. 5B). Results showed that genes with dual modifications displayed the highest expression levels, while lower expression levels were observed with a single feature. Notably, transcript levels of genes occupied only with Pol II were significantly higher than those only featured with ACR (Fig. 5B). Therefore, Pol II occupancy is more accurate in describing gene transcriptional activity than ACR. We also found reduced Pol II occupancy in the proximal regions under light compared to dark conditions, revealing that light inhibits genome-wide transcription activity during seedling photomorphogenesis (Fig. 5, C and D).

Since Pol II is directly involved in the gene transcription, we would like to know the Pol II occupancy in response to IAA and ABA. Then, we conducted the Pol II ChIP-seq of the rice seedlings treated with the exogenous IAA and ABA at 24 h, respectively. To our surprise, the Pol II distribution pattern across genomic structures was greatly altered after spraying with exogenous IAA， in which about 31% peaks were identified in the 3′UTR regions accompanied with reduced density in the exon and distal intergenic regions after the spraying (Fig. 5C). This dramatic increase in Pol II density around TES (Fig. 5D) probably suggested enhanced transcription elongation and termination, which subsequently increased the gene transcription activity in response to exogenous IAA. Furthermore, this was in line with the increased chromatin accessibility density under IAA treatment (Supplementary Fig. S1B). However, the effect of exogenous ABA in altering the Pol II occupancy was not as dramatic as IAA (Fig. 5D). Next, to better decipher the Pol II binding after IAA treatment, we assigned the differential Pol II binding sites to genes. The Kyoto Encyclopedia of Genes and Genomes analysis of these genes suggested that IAA treatment significantly activated Pol II binding on pathways of, for instance, the plant hormone signal transduction, photosynthesis, carbon fixation in photosynthetic organisms, and circadian rhythm in plants (Supplementary Fig. S5). Specifically, genes involved in the auxin-related pathway, such as OsGH3.2 and OsSAUR11, demonstrated higher Pol II and ATAC density under IAA treatment, further positively associating with increased transcript levels (Fig. 5E). All these together suggested the positive roles of exogenous IAA in promoting transcription activity, while no dramatic change of gene transcription activity was identified after the ABA treatment.

## Discussion

Open chromatin reorganization occurs during major developmental phase transitions in plants (Wu et al. 2022). However, chromatin accessibility dynamics and active transcriptional activity during the light-mediated rice seedling photomorphogenesis, as a common and crucial event during the rice development, are still poorly understood. Here we report the epigenomic and transcriptional dynamics underlying rice seedling photomorphogenesis and uncovered chromatin accessibility profiling of rice seedling in response to IAA and ABA. First, our results indicate that chromatin accessibility plays an important role in seedling photomorphogenesis through the complex reprogramming of chromatin state, particularly sorted into 11 clusters. Surprisingly, more than half of these clusters showed reduced chromatin accessibility under light, indicating that repressed chromatin accessibility was also important for the rice photomorphogenesis. In contrast, light indeed activates the chromatin state of those genes involved in photosynthesis. To further explore the roles of chromatin accessibility in mediating chloroplast biogenesis and development during photomorphogenesis, we retrieved the ChIP-seq datasets of 2 rice GLK genes, which are the regulators of chloroplast development. The transcript levels of GLKs are stable from dark to light, while the chromatin accessibility and transcript levels of its targeted genes involved in the photosynthesis process were increased (Fig. 3E and Supplementary Fig. S4). This probably indicates that GLK binding is associated with specific active enhancers in a light-dependent manner to regulate expression of its targets.

ACRs can contain regulatory elements that serve as binding sites for TFs (Huang et al. 2022; Huang et al. 2023); therefore, accessibility changes are considered to be accompanied with gene expression changes. However, we noted the limited overlap between DACRs and DEGs. This further indicated that gene expression is controlled by various layers beyond chromatin accessibility, including DNA methylation or histone modifications (Bulger and Groudine 2011; Wang et al. 2020b; Wang et al. 2022). Besides, many DACRs are located in enhancers, silencers, or other distal regulatory elements (Bulger and Groudine 2011), which may regulate multiple genes or remain inactive under current conditions, thus failing to reflect gene expression.

In this study, we demonstrated that GLKs contribute to rice photomorphogenesis through binding on light-responsive opened chromatin regions. However, the light-responsive TFs of HY5 could also play important roles during the rice photomorphogenesis process. We found that among the 3 homologs of *Arabidopsis* HY5 (AtHY5) in rice (Nijhawan et al. 2008; Burman et al. 2018), only the expression of OsbZIP18 and OsbZIP48 showed positive response to light (Supplementary Fig. S6), while OsbZIP1 was not significantly changed under light. Therefore, OsbZIP18 and OsbZIP48 are the light-responsive TFs, in this study, that potentially play essential roles during the rice photomorphogenesis process. In addition, the PIF family genes in rice (*Oryza sativa*) are known as OsPILs with 6 members, including OsPIL11–OsPIL16 (Nakamura et al. 2007; Sun et al. 2020). It is reported that OsPIF14 (known as OsPIL14, Os07g0143200) plays a crucial role in cross-talk between light and stress signaling (Cordeiro et al. 2016). However, the expression of OsPIF14 was not significantly altered by the light treatment (Supplementary Fig. S6). OsPIL13 (LOC_Os03g56950) was verified to be responsible for the pale-green phenotype in rice (Sakuraba et al. 2017), and it was upregulated under light in this study. This indicates its positive roles for the photomorphogenesis process in rice.

Phytohormones profoundly affect plant growth and development. Auxin is remarkably involved in the regulation of most plant growth and development (Weijers and Wagner 2016), while ABA is frequently defined as a “stress hormone” with roles in the regulation of biotic and abiotic stress responses and also plays roles in plant growth and development under nonstress conditions (Vishwakarma et al. 2017; Yoshida et al. 2019). Auxin and ABA are critical for plant development and stress response, while the chromatin accessibility in rice seedlings mediated by exogenous IAA and ABA is still obscure in rice. In this study, we applied the exogenous IAA and ABA to the seedlings under light at 24 h and investigated the chromatin accessibility. Much more opened chromatin was observed under the application of exogenous IAA than those of ABA (Fig. 4, A, B, and E), indicating the active role of IAA and the repressive role of ABA in regulating chromatin state. The Pol II ChIP-seq data further demonstrated the positive roles of IAA in regulating gene transcription; however, this was not impressive with regard to the ABA treatment (Fig. 5D). Therefore, our data provides evidence of how IAA and ABA regulate chromatin state, thus controlling gene transcription in rice seedling.

Overall, our results revealed a comprehensive understanding of rice seedling photomorphogenesis both in the epigenetic and transcriptional levels, as well as provided valuable resources for studying the roles of cis-regulatory elements in the regulation of hormone responses.

## Methods

### Plant materials and sampling

The rice cultivar of YD6 (*O. sativa* Indica) was germinated under dark conditions in a growth chamber (28 °C) at the Chinese University of Hong Kong, Hong Kong, China. After 3 d of germination in darkness, the germinated rice plants were placed in a growth chamber (BPC600H, Fujian Jiupo Biotechnology Co., Ltd, China) with (28 °C) under light conditions at the Chinese University of Hong Kong, Hong Kong, China. Seedlings at 3 d under dark conditions and seedlings under light conditions after 8 and 24 h were sampled for the following experiments.

### Auxin- and ABA-treated rice seedlings and sampling

Seedlings under light conditions at 23 h and 30 min were immediately sprayed with 100 *μ*ᴍ IAA (dissolved in 1% DMSO), 100 *μ*ᴍ ABA (dissolved in 1% DMSO), and 1% DMSO for 30 min respectively, according to the previously reported method (Galli et al. 2018). Then the treated seedlings were sampled for the following experiments.

### ATAC-seq library construction

Freshly collected seedlings with 2 biological replicates were immediately chopped with a razor blade for 5 min in 100 *μ*L lysis buffer [20 mm MES, pH 5.7, 0.6 m mannitol, 10 mm MgCl_2_, 10 mm KCl, 0.1% 2-mercaptoethanol and 0.2% Triton X-100, 1× PI (protease inhibitor)] on ice (2 biological replicates were performed) according to our previous work (Wang et al. 2022). Thereafter, the chopped slurry was washed with 1 mL lysis buffer in a 1.5 mL tube and incubated for 5 min on ice with gentle shaking. The mixture was filtered through a 40 *μ*m filter into a new tube, followed by centrifugation at 500 g for 8 min (4 °C). Then, we resuspended the precipitation with 150 *µ*L wash buffer (20 mm Tris-HCl, pH 7.8, 10 mm MgCl2, 60 mm KAc, pH 5.6). The above-washed 50,000 nuclei were then tagged with TS-Tn5 at 37 °C for 30 min following the kit's instructions (Vazyme, TD501). After tagging, the integration products were purified using DNA Purification Kit (ZYMO, D4004) and then amplified using Q5 high-fidelity DNA polymerase (New England Biolabs, M0491S) for 10 to 15 cycles. The libraries were sequenced on Illumina HiSeq X Ten with paired-end reads of 150 bp.

### ATAC-seq data analysis

We aligned ATAC-seq reads to *O. sativa* (MSU7.0) reference genome using Bowtie2 (version 2.3.2.) (Langmead and Salzberg 2012). For paired-end 150 bp reads, 100 bp sequences at the 3′ end of the reads were trimmed with the parameter “-3 100.” SAMtools (version 1.9) (Li et al. 2009) was used to filter the low-quality and duplicated reads using the parameters “-F 4 -q 20.” The bigwig files were generated by deepTools (version 2.5.3) (Ramirez et al. 2016). MACS2 was used for ATAC peak calling. We determined the peaks with high reproducibility scores shared between the 2 biological replicates by using the irreproducibility discovery rate (IDR). The differential binding events were identified using the R package “DiffBind.”

### ATAC data footprint analysis

The position weight matrix (PWM) of known rice TFs was obtained from the PlantPAN3.0 database. For ATAC-seq footprint analysis, we used the TOBIAS pipeline following the recommended workflow (Bentsen et al. 2020). The potential open chromatin region binding by the TFs was identified.

### RNA-seq and data analysis

Total RNA was isolated using Plant RNA Extraction Kit (Qiagen, cat. no. 74904) with 2 biological replicates following the manufacturer's instructions, and genomic DNA was removed with the RNase-free DNase Set (Qiagen, cat. no. 79254). Messenger RNAs were isolated with oligo d(T)25 magnetic beads (New England Biolabs, cat. S1419S) and used for preparing the Illumina library. The libraries were sequenced on an Illumina HiSeq X Ten with paired-end reads of 150 bp.

RNA-seq reads were trimmed by seqtk with parameters “trimfq -e 100” and then mapped to rice reference genome using Hisat2 with default parameters. Reads were counted by “HTseq-count” (version 0.11.0, https://htseq.readthedocs.io/en/release_0.11.1/) with parameters “–format = bam –stranded = no –type = mRNA –idattr = Parent.” Fragments per kilobase per million (FPKM) values were calculated by Cufflinks (C. Trapnell et al. 2012). Differential gene expression was performed using R package DESeq2. Genes were considered to be differentially expressed if they showed at least 2-fold difference with an adjusted *P*-value of <0.01.

### ChIP-seq library construction and data analysis

The procedure was followed with our previous work (Wang et al. 2022). Briefly, seedlings with 2 biological replicates were cross-linked with 1% (w/v) formaldehyde in PBS buffer for 30 min, and glycine was added (the final concentration is 0.1 M) to stop the reaction. The cross-linked samples were ground into fine powder in liquid nitrogen. The fine powder was then added with 10 ml nuclei isolation Buffer 1 (10 mm Tris-HCl, pH 8.0, 1 mm EDTA, 0.25 M sucrose, 1% Triton X-100, PI) in a 15 ml tube, which was followed with shaking at 80 rpm on ice for 10 min. Then, the mixture was filtered through a 40 *μ*m filter twice. The filtered supernatant was then centrifuged for 10 min at 6,000 × *g* (4 °C) to obtain the precipitation. The precipitate was then resuspended with Buffer 2 (10 mm Tris-HCl, pH 8.0, 1 mm EDTA, 1% Triton X-100, 1x PI) followed by centrifuging for 10 min at 6000 g (4 °C). We removed the supernatant and resuspended it in Buffer 2 again, followed by centrifugation to obtain the precipitation. Next, 100 *u*L of sonication buffer (10 mm Tris-HCl, pH 8.0, 1 mm EDTA, 0.2% SDS, 1× PI) was added to the precipitate and mixed well for sonication (30 cycles of 30 s on and 30 s off on a Diagenode Bioruptor). Tubes were centrifuged at 10,000 × *g* for 5 min, and supernatants were transferred to new tubes. At this point, ChIP input aliquots were collected.

Dynabeads Protein A (Thermo Fisher Scientific, cat. no. 10002D) were washed with low salt buffer (10 mm Tris-HCl, pH 8.0, 1 mm EDTA, 150 mm NaCl, 1% Triton X-100, 1× PI) and then rotated with antibodies (anti-RNA polymerase II CTD repeat YSPTSPS antibody—ChIP Grade, ab26721) at a concentration of 2 *μ*g antibody per 100 *μ*L of ChIP dilution buffer overnight at 4 °C. After binding, the beads were washed with low salt buffer (10 mm Tris-HCl, pH 8.0, 1 mm EDTA, 150 mm NaCl, 1% Triton X-100, 1× PI), high salt buffer (10 mm Tris-HCl pH 8.0, 1 mm EDTA, 500 mm NaCl, 0.5% Triton X-100, 1× PI), and 10 mm Tris-HCl (pH 8.0) twice. The immunoprecipitated DNA was tagged with TS-Tn5 (Vazyme, TD-501) at 37 °C for 30 min. They were then washed with low salt buffer, high salt buffer, and 10 mm Tris-HCl. After being reverse cross-linked and treated with proteinase K, DNA was purified with DNA recovery beads. The recovered DNA was then amplified following 72 °C for 2 min and 98 °C for 30 s; then 10 to 15 cycles of 98 °C for 15 s, 63 °C for 30 s, and 72 °C for 30 s; and once at 72 °C for 1 min using TruePrep Index Kit V2 for Illumina (Vazyme, cat. no. TD202). PCR products were purified with AMPure beads to remove primers. The libraries were then sequenced on Illumina X Ten platforms in PE150 mode.

ChIP-seq reads were first aligned to *O. sativa* (MSU7.0) reference genome using Bowtie2 (version 2.3.2.) (Langmead and Salzberg 2012). We used SAMtools (version 1.9) (Li et al. 2009) to filter the low-quality and duplicated reads using the parameters “-F 4 -q 20.” We applied MACS2 with parameters “-g 2e8 –nomodel -q 0.01” to call peaks that were normalized with the input control.

### Interplay of GLK ChIP-seq data with ATAC-seq

We retrieved 2 rice GLK ChIP-seq data from NCBI database under accession code PRJNA682315GSE220115 and deployed MACS2 to call the peaks that were normalized with its input control. These peaks were further annotated as ATAC-seq peaks described above.

### Genomic distribution of peaks

The gene annotation file (Osativa_323_v7.0.gene.gff3) was used for the genomic distribution of peaks. Two kb upstream of the transcription start site (TSS) of annotated protein-coding genes was defined as promoters. The gene body was defined as the region between the TSS and the transcription termination site (TES). Downstream regions refer to >300 bp from the TES, while the rest of the genome was annotated as distal intergenic regions. Peaks were assigned to each category using their peak summits.

### Chlorophyll content analysis

About 1 g seedling was used for chlorophyll extraction with 3 biological replicates using 20 mL extraction buffer (ethanol:acetone:ddH2O = 9:9:2 v/v) at room temperature for 48 h under dark conditions. Then the extracted solution was measured with absorbance at 645 and 663 nm. The chlorophyll content was calculated by following the previously reported method (Richardson et al. 2002).

### GO analysis

Functional GO enrichment analysis was performed by web-based toolkit for the agricultural community agriGO v2.038 (http://systemsbiology.cau.edu.cn/agriGOv2/). GO terms with a false discovery rate (FDR) < 0.05 were considered significantly enriched.

### Accession numbers

Sequence data from this article can be found in the NCBI database under accession numbers GSE303225 and GSE303510.

## Supplementary Material

kiaf438_Supplementary_Data

## Data Availability

Sequence data from this article can be found in the NCBI database under accession numbers of GSE303225 and GSE303510.

## Supplementary data

The following materials are available in the online version of this article.

**Supplementary Figure S1.** Overview of chromatin accessibility among different samples.

**Supplementary Figure S2.** Correlation of DACRs and DEGs.

**Supplementary Figure S3.** Targets of GLKs involved in the photosynthesis process.

**Supplementary Figure S4.** GO analysis of ABA- or IAA-altered ACRs.

**Supplementary Figure S5.** KEGG analysis of IAA-activated genes revealed by RNA Pol II binding.

**Supplementary Figure S6.** Expression pattern of the *Arabidopsis* homologs of HY5 as well as its targets in rice.

**Supplementary Table S1.** Data summary of ATAC-seq and ChIP-seq.

**Supplementary Table S2.** ATAC-seq peak clusters.

**Supplementary Table S3.** Transcript levels of all the samples.

**Supplementary Table S4.** TF binding motifs with differential ATAC-seq footprint scores.

**Supplementary Table S5.** TF binding motifs showed significantly different ATAC-seq footprint scores in different comparisons.

**Supplementary Table S6.** ATAC-seq peak clusters altered by ABA and IAA.
