# Supplementary material for: Altered chromatin accessibility in light and hormone responses of rice seedlings
Source: Plant Physiol. 2025 Sep 26;199(2):kiaf438. doi: 10.1093/plphys/kiaf438 (PMC12530099; doi:10.1093/plphys/kiaf438)
Supplement: kiaf438_Supplementary_Data [file kiaf438_supplementary_data.zip › Supplementary Figures.pdf]

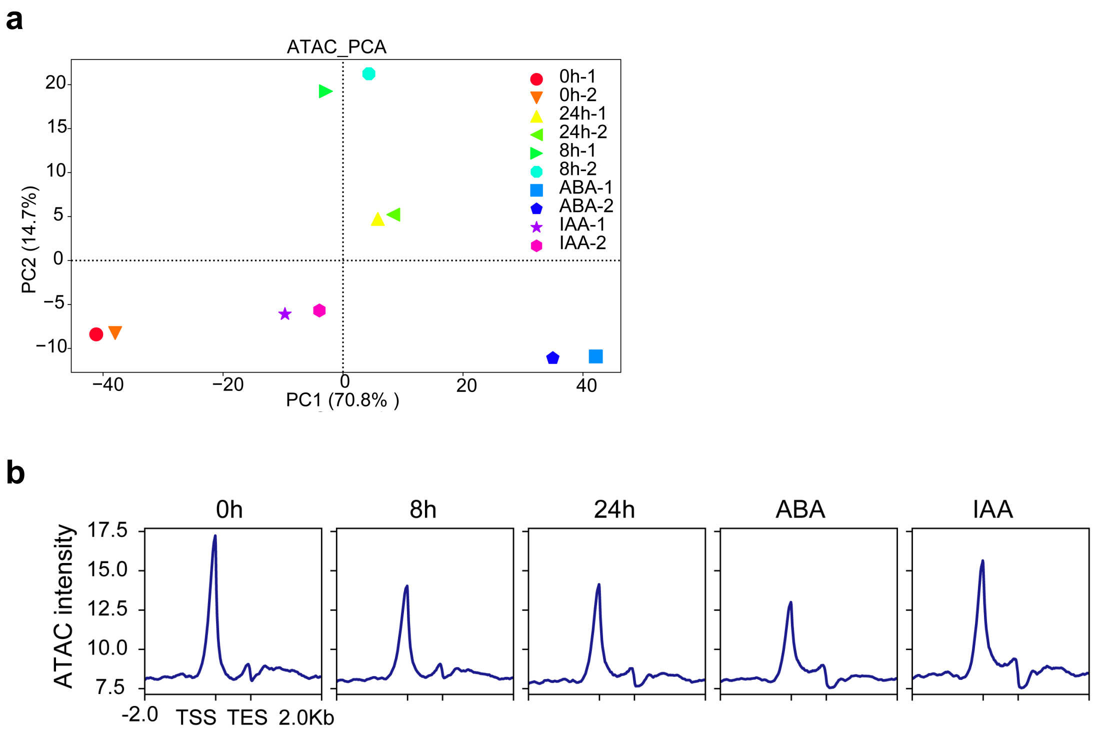

Supplementary Figure S1. Overview of chromatin accessibility among different samples. a, Principal-components plots (PCA) of ATAC-seq dataset. Color code is shown. Each dot represents one sample. b, Normalized ATAC density of all the samples within gene coding regions. Seedlings under dark was defined as the first time point (0h), seedlings exposed to light for 8 hours (8h) and 24 hours (24h) were sampled as the second and third time points, respectively. Short-term auxin (IAA) and ABA treatment on rice seedlings at 24 hours (100 µM IAA and 100 µM ABA for 30 min) were conducted. TSS transcription start sites. TES, transcription end sites.

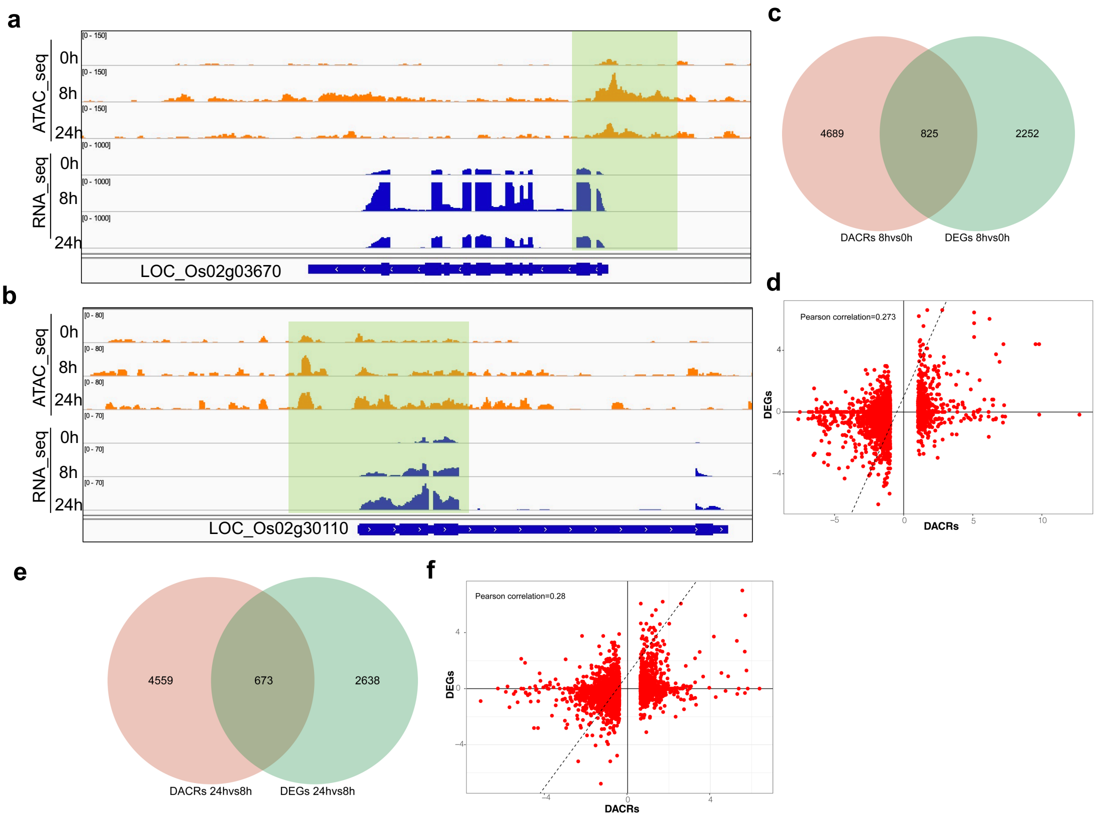

Supplementary Figure S2. Correlation of DACRs and DEGs

a and b, IGV showing chromatin status on gene of LOC_Os02g03670 belonged to cluster C2 (a), and gene of LOC_Os02g30110 in cluster C5 (b). c and d, Venn diagram showing number of differential ACRs (DACRs) overlapped with DEGs (c) and its Pearson correlation between DACRs and DEGs (d) in the comparison between 8h and 0h. e and f, Venn diagram showing number of differential ACRs (DACRs) overlapped with DEGs (c) and its Pearson correlation between DACRs and DEGs (d) in the comparison between 24h and 8h.

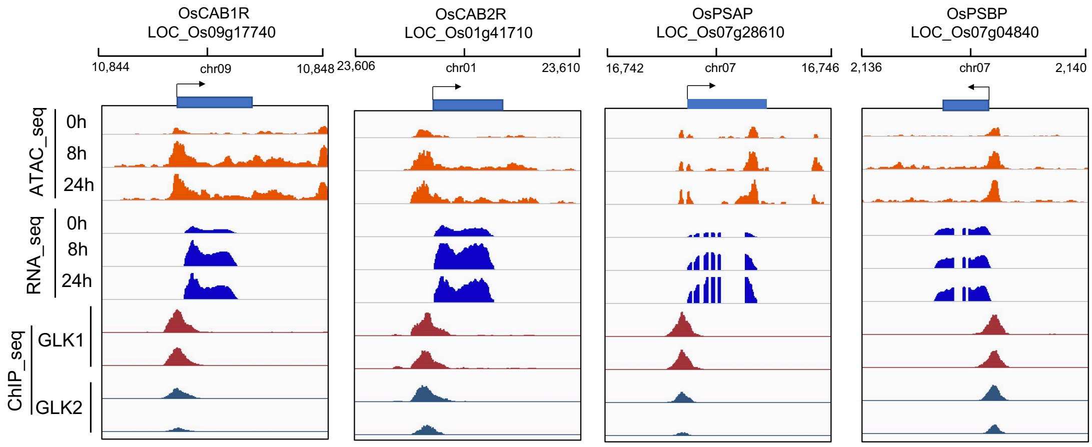

Supplementary Figure S3. Targeted of GLKs that involved in the photosynthesis process. Chromatin accessibility, transcript level and GLK-ChIP signals were showed in IGV.

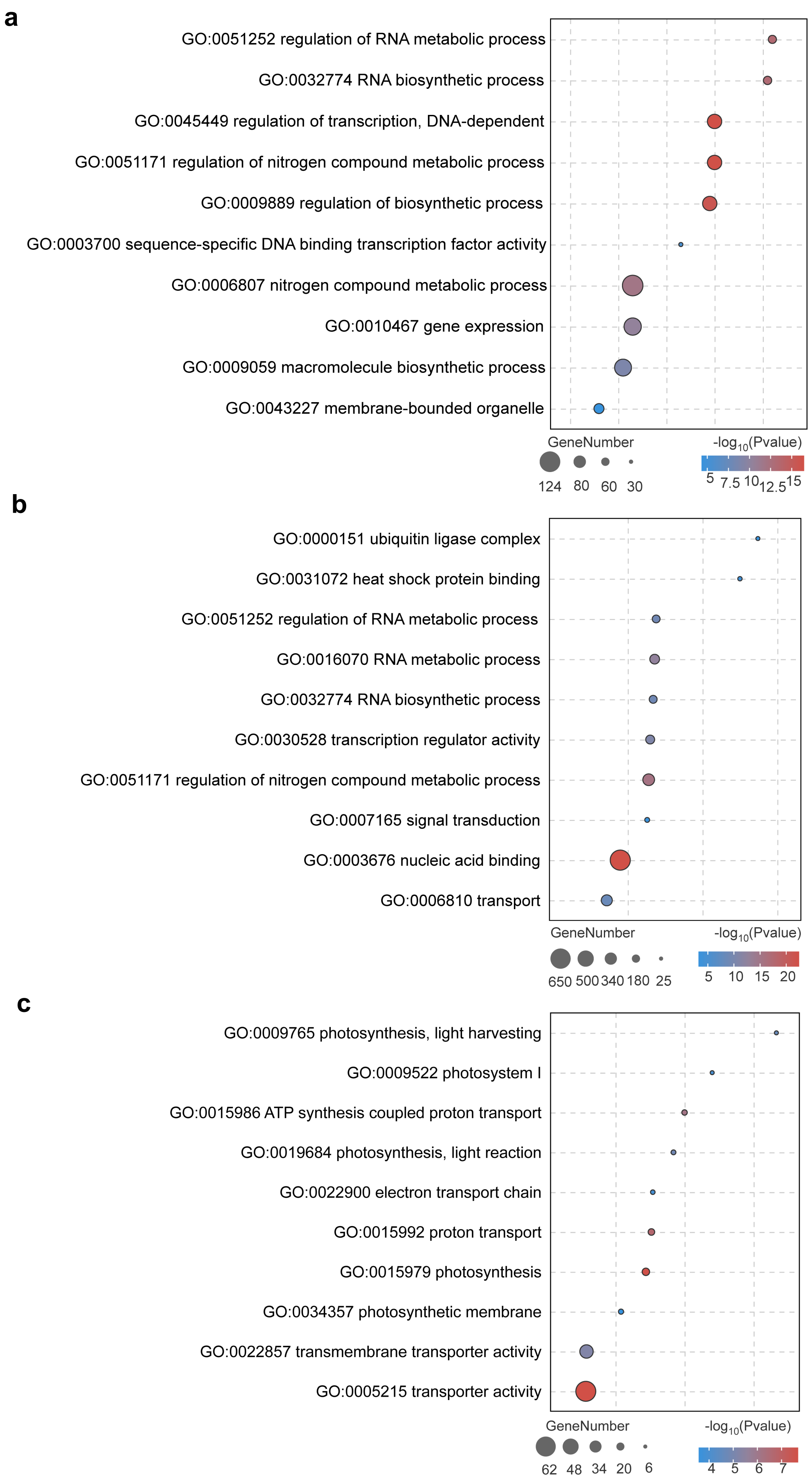
a
GO:0051252 regulation of RNA metabolic process
GO:0032774 RNA biosynthetic process
GO:0045449 regulation of transcription, DNA-dependent
GO:0051171 regulation of nitrogen compound metabolic process
GO:0009889 regulation of biosynthetic process
GO:0003700 sequence-specific DNA binding transcription factor activity
GO:0006807 nitrogen compound metabolic process
GO:0010467 gene expression
GO:0009059 macromolecule biosynthetic process
GO:0043227 membrane-bounded organelle
GeneNumber
124 80 60 30
-log10(Pvalue)
5 7.5 10 12.5 15
b
GO:0000151 ubiquitin ligase complex
GO:0031072 heat shock protein binding
GO:0051252 regulation of RNA metabolic process
GO:0016070 RNA metabolic process
GO:0032774 RNA biosynthetic process
GO:0030528 transcription regulator activity
GO:0051171 regulation of nitrogen compound metabolic process
GO:0007165 signal transduction
GO:0003676 nucleic acid binding
GO:0006810 transport
GeneNumber
650 500 340 180 25
-log10(Pvalue)
5 10 15 20
c
GO:0009765 photosynthesis, light harvesting
GO:0009522 photosystem I
GO:0015986 ATP synthesis coupled proton transport
GO:0019684 photosynthesis, light reaction
GO:0022900 electron transport chain
GO:0015992 proton transport
GO:0015979 photosynthesis
GO:0034357 photosynthetic membrane
GO:0022857 transmembrane transporter activity
GO:0005215 transporter activity
GeneNumber
62 48 34 20 6
-log10(Pvalue)
4 5 6 7

Supplementary Figure S4. GO analysis of ABA or IAA altered ACRs. a, GO analysis of genes associated with ABA induced ACRs. b, GO analysis of genes associated with IAA induced ACRs. c, GO analysis of genes associated with IAA repressed ACRs.

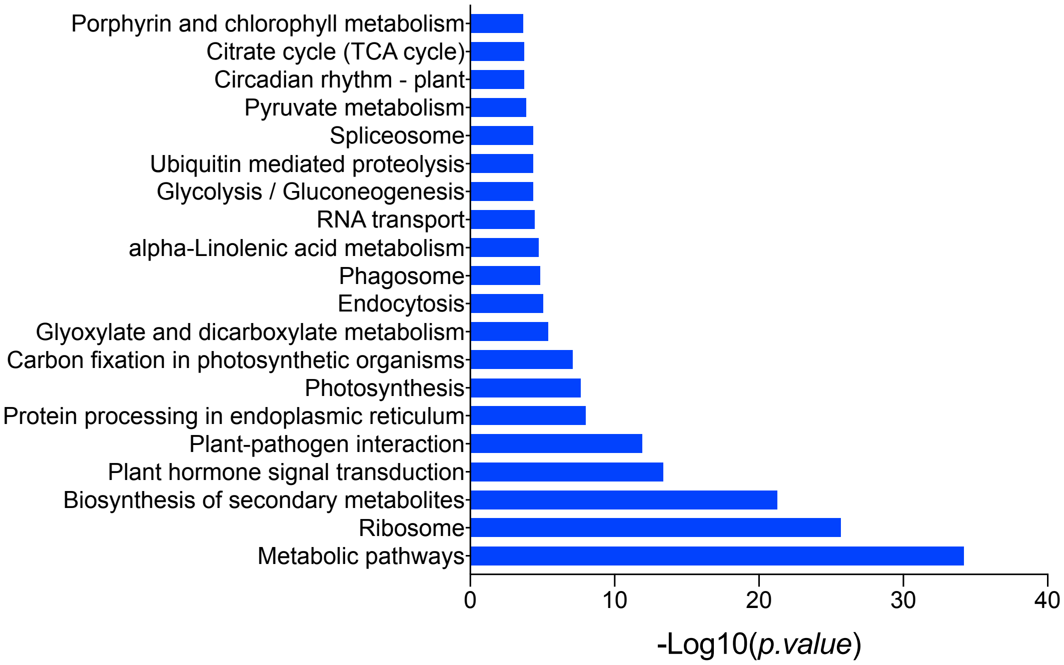

Supplementary Figure S5. KEGG analysis of IAA activated genes revealed by RNA Pol II binding.

KEGG analysis of these genes occupied by Pol II treated with exogenous IAA.

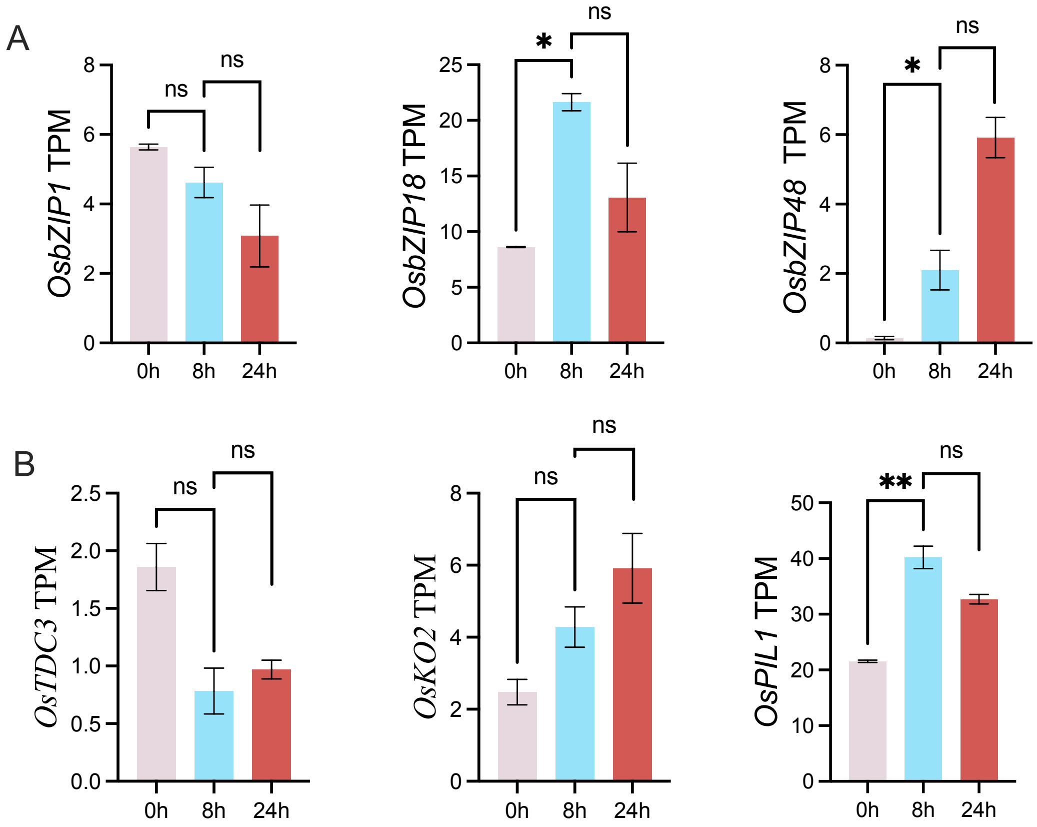

Supplementary Figure S6. Expression pattern of the *Arabidopsis* homologs of HY5 as well as its targets in rice.

a, Expression levels of three homologs of *Arabidopsis* HY5 (AtHY5) *OsbZIP1* (Os01g07880), *OsbZIP18* (Os02g0203000), and *OsbZIP48* (LOC_Os06g39960) at three stages. b, Expression levels of OsbZIP18 target *OsTDC3*, OsbZIP48 target *OsKO2* and *OsPIL1*(also named as *OsPIL13*) at three stages. Data are means ± SD. n = 2. Seedlings under dark was defined as the first time point (0h), seedlings exposed to light for 8 hours (8h) and 24 hours (24h) were sampled as the second and third time points, respectively. The *p*-value was determined by a student t-test. ns=not significant; * represents *p*-value less than 0.05; ** represents *p*-value less than 0.01.
